# Extracellular Vesicles in the Oviduct: Progress, Challenges and Implications for the Reproductive Success

**DOI:** 10.3390/bioengineering6020032

**Published:** 2019-04-12

**Authors:** Carmen Almiñana, Stefan Bauersachs

**Affiliations:** 1 Genetics and Functional Genomics Group, Vetsuisse Faculty, University of Zurich, 8057 Zurich, Switzerland; stefan.bauersachs@uzh.ch; 2 UMR85 PRC, INRA, CNRS 7247, Université de Tours, IFCE, 37380 Nouzilly, France

**Keywords:** extracellular vesicles, exosomes, microvesicles, oviduct, gamete/embryo-oviduct interactions

## Abstract

The oviduct is the anatomical part of the female reproductive tract where the early reproductive events take place, from gamete transport, fertilization and early embryo development to the delivery of a competent embryo to the uterus, which can implant and develop to term. The success of all these events rely upon a two-way dialogue between the oviduct (lining epithelium and secretions) and the gametes/embryo(s). Recently, extracellular vesicles (EVs) have been identified as major components of oviductal secretions and pointed to as mediators of the gamete/embryo-maternal interactions. EVs, comprising exosomes and microvesicles, have emerged as important agents of cell-to-cell communication by the transfer of biomolecules (i.e., mRNAs, miRNAs, proteins) that can modulate the activities of recipient cells. Here, we provide the current knowledge of EVs in the oviductal environment, from isolation to characterization, and a description of the EVs molecular content and associated functional aspects in different species. The potential role of oviductal EVs (oEVs) as modulators of gamete/embryo-oviduct interactions and their implications in the success of early reproductive events is addressed. Lastly, we discuss current challenges and future directions towards the potential application of oEVs as therapeutic vectors to improve pregnancy disorders, infertility problems and increase the success of assisted reproductive technologies.

## 1. Introduction

Maternal interactions with gametes and embryo(s) are considered as the basis for reproductive success [1,2]. Transcriptomic and proteomics studies have demonstrated that these interactions start in the oviduct, the anatomical tube connecting the ovary and the uterus [3,4,5,6,7]. The oviduct provides the optimal microenvironment for gamete maturation, transport, and the guidance of spermatozoa towards the oocyte(s) leading to successful fertilization, as well as supporting very early embryo development [8,9]. Moreover, the oviduct has been pointed to as a modulator of the epigenetic landscape of the embryo [10]. Increasing evidence from human and animal models indicates that the use of assisted reproductive technologies (ARTs), such as intracytoplasmic sperm injection or in vitro fertilization, which bypasses these gamete/embryo-oviductal interactions, are associated with genomic imprinting disorders [11,12]. These findings call for a better understanding of the complex oviductal environment that can be mimicked by embryologists and reproductive biologists in the laboratory, to increase the success of ARTs.

Considering the complex role of the oviduct in the early reproductive events, efforts have been directed to unravel the composition of the oviductal milieu under different reproductive scenarios: under different hormonal regulation or in response to spermatozoa/embryos. Having a look into the literature, studies can be divided in two different approaches: (1) focusing on oviductal epithelial cells (OEC) [4,7,13,14,15,16,17], and (2) focusing on the oviductal fluid/secretions [18,19,20,21]. As a result, a large number of oviductal components, including hormones, growth factors and their receptor, enzymes, have been detected in oviduct and/or oviductal fluid. The list of oviductal proteins is increasing every year with the use of more sensitive mass spectrometer instruments. However, the question that arises is how all these individual components may interact with spermatozoa/embryos or be taken up by them to exert a functional effect.

Recently, oviductal extracellular vesicles (EVs) have been identified as major components of the oviductal fluid and potential mediators of the gamete/embryo maternal interactions [22,23,24]. EVs, referred to as exosomes (40–100 nm) and microvesicles (>100 to 1000 nm), are present in most body fluids and have emerged as a new way of cell-to-cell communication [25]. EVs contain a repertoire of bioactive molecules such as proteins, mRNAs, small ncRNAs, lipids and also, genomic DNA [25]. Interestingly, this molecular cargo is protected from extracellular degradation or modification [26]. Besides, the cargo reflects the physiological and pathological status of the cell of origin [25]. Furthermore, the molecular components can be transferred to recipient cells and exert a functional effect in the recipient cell (reviewed by [27]). These intrinsic characteristics of EVs make them great biomarkers of infertility/pregnancy failure and potential therapeutic agents in ART and fertility treatments [26,28,29,30]. 

To date, oviductal EVs (oEVs) have been identified in the oviductal fluid of different species (mouse [22,31], cow [23,24], woman: [32], hen [33], bitch [34] and turtle [35]). However, little is known about the oEVs molecular content and associated functions, compared to other maternal reproductive EVs such as uterine EVs or follicular fluid EVs. Few studies have shown that oEVs can exert a functional effect on sperm, by enhancing sperm fertilizing ability [22,32] and as well as on the embryo, by improving embryo development and cryoresistance [23,24] and enhancing the efficiency of embryo transfer by increasing the birth rate [36]. While only three studies have brought some light into the oEVs molecular cargo [31,37]. The scant information on oEVs emphasize the need for deeper analyses of their molecular composition and functional effects, which will bring a better understanding of the oEVs contribution to the gamete/embryo-oviduct dialog and their implications in the reproductive success. 

The objective of this review is to highlight recent progress and challenges in oEVs research to increase our understanding about the role of oEVs in the oviduct and their implications in the reproductive success, since gametes and embryos are surrounded by oEVs during a short time frame but with huge consequences persisting into adulthood.

## 2. Studies on Oviductal EVs

Despite the fact that EVs were discovered almost four decades ago [38], oEVs were identified quite recently in the murine oviductal fluid by Al-Dossary and colleagues [22], who dubbed them “Oviductosomes”. This term was used to align with the terms used for EVs identified in the male reproductive tract (epididymosomes and prostasomes [39,40]) and in the female tract (uterosomes [41]). Oviductal EVs vary in origin and size and according to that have been classified into two categories: (1) exosomes arising from endosomes with 40–100 nm in size (or 30–200 nm, depending on literature); and (2) microvesicles budding from the plasma membrane and with a size between 100 nm–1 μm. Current state-of-the-art analysis of the biology of exosomes and microvesicles can be found in detail in van Niel et al., [27] and related to the reproductive field in Simon et al. [26]. To clarify, the term oviductosomes or oEVs refers to both exosomes and microvesicles, and in the present review we will refer to them as oEVs. 

Considering the existing literature on oEVs, it calls our attention that the number of publications focused on oEVs is lower than for other EVs derived from the maternal tract, such as uterine EVs and follicular fluid EVs. The low number of studies on oEVs could be due to: (1) the difficulty in collecting oviductal fluid samples, since to obtain it, animals need to be slaughtered or subjected to surgery; and/or (2) the underestimated role of the oviductal milieu [42,43] on sperm/embryo and for extension oEVs. Compared to the uterus, embryo spend only a short time in the oviduct (from two to six days depending on the species). Furthermore, embryos can be produced in vitro, develop up to day seven and then establish a pregnancy after embryo transfer, despite their quality is inferior than in vivo embryos [44].

Before entering into the discussion of current studies on oEVs, we consider it important to look at different isolation and purification techniques used by oEVs studies. It is known that different isolation methods can lead to the collection of different populations of exosomes/microvesicles (MVs) [45] and also that the methodology affects the exosome yield and composition of the molecular cargo in terms of omics profiles of exosome populations [46]. It is worth mentioning that all oEVs studies found in current literature used ultracentrifugation/serial centrifugation as the preferential method to isolate EVs, in contrast to other maternally-derived EVs or EVs from different biological fluids (urine, serum, saliva, etc.), where different methods were used (density gradient centrifugation, polymer-based precipitation, and immunoaffinity as well as commercial kits such as ExoQuick™ precipitation or the Total Exosome Isolation™ precipitation solution). Detailed comparative analyses among EVs isolation methods have been performed and reviewed in the literature [47,48,49,50,51,52,53,54,55] and are not the focus of this review. Here, we will focus on the pros and cons of the use of ultracentrifugation since it has been the method used in all studies of oEVs so far.

The selection of ultracentrifugation as the common method among all oEVs could be random, or could be due to the fact that most publications have aimed to analyse the functional effects of EVs on gametes/embryos. Ultracentrifugation is considered the gold standard method for exosome isolation and the most widely applied in EVs studies [46,56]. It is the preferred method to obtain EVs for downstream analysis and for performing further experiments on biological functions, despite the low exosome yield and contamination with impurities. As Lötvall and colleagues pointed out, some density gradients or immunoaffinity methods often used for EV isolation, with better yield and exosome purity, may alter or impede functional tests [57]. So far, ultracentrifugation seems the preferred method for subsequent use of EVs pellet in flow-cytometry, proteomics, lipidomics, RNA, and biological studies at the same time. Furthermore, the use of ultracentrifugation can help to preserve the integrity and the biological activity of isolated EVs as well as the sterility, which is an important point for functional experiments of oEVs with gametes and embryos. Additionally, recent studies have pointed out that it is important to keep the exact same protocol with differential centrifugation steps, when comparative studies on EVs are performed. Since the different types of rotors and the specific tubes used for these rotors can affect the k-factor, as well as centrifugation time, producing 2–3-fold differences in pellet yield [47].

Regarding oEVs characterization, most studies have chosen as least two methods, one based on morphology (or single vesicle analysis such transmission electron microscope (TEM), or nanoparticle tracking analysis (NTA) and one molecular, based on protein markers (Western blot, flow cytometry), as suggested by the International Society for Extracellular Vesicles guidelines (ISEV) (on MISEV2014, [57]). However, regarding the criteria for the selection of exosomal markers found in published oEVs studies, there is not a common approach. Different exosomal markers have been used in oEVs studies such as: tetraspanins CD9 and CD81; heat shock proteins HSP70 (also known as HSPA1A) and HSC70 (also known as HSPA8); as well as TSG101 and actin-linking ezrin-radixin-moesin (ERM) proteins (see Table 1). We believe that the lack of consensus reflects the current state of EVs research, since so far there is not a defined list of EV-specific “markers” that distinguish different subtypes of EVs, only different EVs-enriched proteins have been proposed by MISEV2014 and updated recently in more detail in MISEV2018 [55]. Moreover, the limited amount of oEVs as starting material makes it difficult to perform a deep characterization as recommended by MISEV2018 [55], leading researchers to limit the characterization experiments in favour of further omics analysis of the content and/or functional experiments in their studies.

The increasing research in EVs demands urgent standardized strategies for EVs isolation with high purity and high exosome yield as well as for EVs-specific “markers” to sufficiently characterize the different EV populations. Isolation strategies and specific markers will need to be re-adjusted for oEVs in a practicable manner. Until then, the conclusions about the molecular cargo and functions of oEVs should be discussed with caution, being aware that under different sampling conditions or different isolation strategies for oEVs, the results might be slightly different. This brings up the need for full reporting of oEVs experiments with detailed descriptions of sampling conditions, characterizations, omics analysis, and functional aspects for each experiment.

In the present review, we attempt to bring together current knowledge of oEVs and their contribution to the maternal gamete/embryo dialogue. For a better understanding of the impact of the existing studies, we have categorized oEVs studies according to three different topics: (1) analysis of oEVs molecular content; (2) oEVs and their functional effects on gametes (sperm and oocyte); and (3) oEVs and their functional effects on the embryo(s) (Table 1). 

### 2.1. Analysis of oEVs Molecular Content 

We have included in this section studies providing an extensive analysis of oEVs content at protein, mRNA or miRNA level. Studies analysing only a unique protein or a panel of a few proteins, mRNAs or miRNAs in oEVs are not included in this section; although they provide important information about the oEVs components, and thus they are discussed in Section 3 of this review (included in Table 1). Considering this, we found in the literature only three publications providing an extensive repertoire of oEVs molecular components so far. Two on bovine oEVs from our laboratory [24,37] and other one on murine oEVs by Fereshteh et al. [31]. In fact, only our study on bovine oEVs across the estrous cycle has performed a systematic study of the oEVs molecular content at different levels using the same samples [37]. In our first study on oEVs, we compared the protein content of oEVs released by OEC in vivo obtained by flushing the oviducts and in vitro obtained from primary culture of OEC [24]. Our study revealed considerable differences in the protein cargo between oEVs from in vivo and in vitro origin. This study led us to three important ideas: (1) that the oEVs may be under the hormonal regulation; (2) that caution needs to be taken when studies are only based on EVs derived from cell lines or primary culture and extrapolated to in vivo EVs biology and function; and (3) the need to further analyse the molecular components of oEVs, e.g., at the level of mRNAs and miRNAs. Recently, we performed the first extensive analysis of oEVs providing their molecular repertoire at protein, mRNA and small ncRNA level [37]. Our results showed the oEVs molecular content is under the hormonal influence of the estrous cycle. In the next section, we will discuss some of the different proteins, mRNAs and miRNAs that changed in their abundance in oEVs across the estrous cycle and their potential functions in the oviduct. 

### 2.2. Oviductal EVs and Their Functional Effect on Gametes

Oviductal EVs were first described for their implications in sperm storage, promotion of capacitation and regulation of acrosome reaction and hyperactivated motility in mice [22]. Al-Dossary and colleagues demonstrated that oEVs carry in their cargo plasma membrane Ca^2+^-ATPase 4 protein (PMCA4), which is the major Ca^2+^ efflux pump in murine sperm [61]. Particularly, the PMCA4a form is predominant in the oviductal fluid compared to uterus and highly abundant during proestrus/estrus compared to other stages of the cycle. Moreover, PMCA4a is involved in the maintenance of Ca^2+^ homeostasis, prevent premature capacitation of the sperm and support sperm acquisition of fertilization potential [22]. Furthermore, it has been shown that deletion of the gene for PMCA4a leads to male sterility due to a loss of progressive and hyperactivated motility [62,63]. Spermatozoa acquire PMCA4a from the oviductal secretions via oEVs as demonstrated after in vitro co-incubation of oEVs with sperm [22]. The mechanism by which oEVs deliver PMCA4a or other cargo to sperm cells is by integrin αvβ3 and α5β-mediated oEVs fusion to the sperm membrane [58].

Recently, oEVs were described in human oviductal secretions for the first time (Bathala et al., 2018). Human oEVs carry also PMCA4 and eNOS which are fertility-modulating sperm proteins [62] and PMCA1, which leads to embryonic lethality in mice when deleted [63]. Bathala et al. [32] suggested that all proteins identified to date in the cargo of murine oEVs with a fertility-modulatory role have a conserved function in human oEVs.

On the other hand, Lange-Consiglio et al. [34] have shown that oEVs can exert also a functional effect on the female gamete, contributing to the oocyte maturation in the bitch. The canine oviductal environment has some unique features that contribute to the oocyte maturation in the oviduct. In the absence of this unique milieu in vitro, the canine oocyte maturation rates are very low. Lange-Consiglio and colleagues [34] showed that the supplementation of in vitro maturation media with MVs from canine oviductal fluid improved significantly canine oocyte maturation rates. Moreover, these authors demonstrated that MVs can be taken up by the cumulus cells and later into the oocyte cytoplasm during in vitro maturation. Furthermore, these authors identified in oviductal cells and MVs three miRNAs (miR-30b, miR-375 and miR-503) with key roles in follicular growth and oocyte maturation. Considering that canine oocyte maturation occurs in the oviduct in contrast to other species, these miRNAs may be transferred from the oviductal cells to the oocytes via MVs to support oocyte maturation. In bovine, these three miRNAs were also found in follicular fluid EVs [64], where the oocyte maturation takes places, while only miR-30b and miR-375 were found in oEVs [37]. Lange-Consiglio et al. [34] suggest that specific MVs cargo may be essential for canine oocyte maturation and thus the supplementation of MVs during canine IVM may have a great impact on the in vitro reproductive technologies for the canine species. 

### 2.3. Oviductal EVs and Their Functional Effect on Embryo(s)

To date only four studies have shown functional effects of oEVs on embryos (Table 1). Almiñana et al. [24] showed that oEVs could be taken up by in vitro produced (IVP) embryos during in vitro culture (IVC). Moreover, this study showed that the oEVs supplementation during IVC improved embryo development in terms of blastocyst yields, hatching rates, in vitro survival and number of cells/embryo. Similar results were obtained by Qu et al. in mice [36] when they compared the effect of oEVs from the murine donor and recipient oviductal fluids on cleavage and blastocyst rate to observe a significant increase in blastocyst rates in oEVs by donors. Additionally, these authors demonstrated that oEVs from donors improved the efficiency of embryo transfer by increasing the birth rate. Besides, Lopera-Vázquez et al. [23] showed that oEVs supplementation exert an effect on embryo quality, by increasing also the number of cells and improving embryo cryosurvival after cryopreservation, although no effect was observed on the blastocyst yield. Furthermore, Lopera-Vásquez and colleagues [59] showed differences in EVs concentration and size in different regions of the oviduct (ampulla and isthmus) and with different functional effects on the embryo. While Qu et al. [36] pointed out differences in oEVs from donors and recipients, with higher concentrations and more protein in oEVs from donors than recipients. 

Differences in the results obtained among studies could be due to two main reasons: (1) the hormonal influences on the oEVs content [37], since samples were collected from animals at different stages of the estrous cycle (early post-ovulatory stage, 1–4 days after ovulation [24] versus mid-luteal phase [23]), and (2) the different origin of oEVs (in vivo oEVs obtained after flushing of the oviducts versus oEVs obtained from conditioned media after primary culture of BOEC [23]), since it has been shown that oEVs from in vivo and in vitro origin have a different molecular cargo [24].

To prove that oEVs could have further effects on IVP embryos, Lopera-Vázquez et al. [59] analysed the embryonic gene expression of IVP embryos cultured with oEVs focusing on a panel of genes. Supplementation with oEVs altered gene expression in the embryo by the down-regulation of *IFNT* and *PLAC8*, genes related to implantation, when compared to the control. Interestingly, oEVs supplementation led to embryos with *IFNT* and *PLAC8* expression patterns more similar to in vivo ones, since *IFNT* and *PLAC8* have been found as down-regulated in in vivo embryos compared to in vitro counterparts [65,66]. On the other hand, Que et al. [36] showed that the supplementation of IVC media with murine oEVs alters the expression of genes related to apoptosis and cell proliferation (*BAX, BCL2, OCT4*) in the embryo. The use of oEVs from donors revealed a lower apoptotic index compared to recipients, suggesting improved embryo competency by repressing apoptosis and promoting differentiation.

Despite differences in the material of origin and isolation protocols, all studies mentioned above showed a positive effect of oEVs supplementation during in vitro culture on the embryo development by improving embryo development and quality [24]; embryo cryosurvival [23], embryonic gene expression [23] as well as birth rates [36]. Moreover, it has been shown that oEVs from isthmus compared to ampulla [59] and from oviducts from donors compared to receptors [36], improved IVP embryos in terms of blastocyst rates and birth rates. These results seem logical and may reflect the physiological needs of the embryo, since the early embryo is located in the isthmus, which sustains its development before migrating into the uterus. Furthermore, the exosome secretion/content seems to be different between donor and recipients despite hormonal treatments [36]. All these studies provide complementary findings regarding the functional effect of oEVs on the early embryo and their fate. Furthermore, they clearly showed that oEVs are an important part of the oviductal secretions with crucial effect on embryo development and therefore could be a good strategy to improve ARTs success.

## 3. Oviductal EVs: Current Knowledge about Their Molecular Content

### 3.1. Oviductal EVs and Proteins

In bovine oEVs, 336 clusters of proteins were identified by mass spectrometry analysis [37]. Among them, we highlight the presence of proteins characteristic for the exosomal proteomics signature with different roles such as: exosome biogenesis (ESCRT-associated: VPS13C, FAM129B, PDCD6IP); intracellular vesicle trafficking and release (DYN-C1LI1; SNAP23, STXBP1); tetraspanins (CD9) and GTPases (RAB1A, RAB2A, RAB7A, RAB11A, RAB5C); exosome sorting (COPA, CLTC, ARF1, RAC1); annexin proteins involved in membrane trafficking and fusion events (ANXA1–8, ANXA10); and heat-shock proteins (HSPs) (HSP90, HSC70) involved in homeostasis and biosynthesis, transport and folding of proteins. Besides, we identified proteins characteristic for the oviduct with key roles during the gamete/embryo-oviduct cross talk such as: OVGP1, ANXA2, HSPA8, HSP90, HSP70, gelsolin and ezrin (discussed in [24]). Some of these proteins were among the 25 most abundant proteins in oEVs, which could exert a functional effect on spermatozoa or embryos when they are taken up via oEVs. Proteins of the HSP family were identified (HSPB1, HSPA8, HSP90AA1 and HSPA1A), which are crucial for fertilization and early embryo development [67] and can exert anti-apoptotic effects and cryoprotection (HSPB1) [68]. Annexin family proteins have also been identified in oEVs in high abundance, and have been suggested to hold sperm in the oviductal reservoir (ANXA2) [69]. Interestingly, some proteins showed different patterns of abundance between post-ovulatory and pre-ovulatory stages of the estrous cycle, maybe adapting to their role on sperm and/or embryos. For example, MYH9 and CLTC were found to be more abundant at pre-ovulatory stages, and potentially involved in the maintenance of sperm viability in the sperm reservoir [70]. While HSPA8, MIF and AQP5 were more abundant at post-ovulatory stages and may contribute to the release of spermatozoa from the sperm reservoir, sperm hyperactivation, and sperm transport to meet the oocyte, fertilization and early embryo development [71].

In murine oEVs, Al-Dossary and colleagues [22] identified a plasma membrane Ca^+2^-ATPase 4 (PMCA4), which is involved in controlling progressive and hyperactivated sperm motility. While Griffiths et al. [72] identified sperm adhesion molecule 1 (SPAM1), contributing to enhance hyaluronic acid-binding ability and cumulus penetration efficiency. Bathala et al. [32] identified the presence of tyrosine phosphorylated sperm proteins that play a critical role in sperm capacitation and that can be delivered to sperm via oEVs. Additionally, these authors identified two proteins, calcium/calmodulin-dependent serine kinase (CASK) and neuronal nitric oxide synthase (nNOS), which interact with PMCA4, as well as PMCA1 which is present in murine oEVs in a functional complex. However, most of the proteins identified in murine oEVs were not identified in bovine oEVs, which could be explained by differences in oEVS isolation protocols, the proteomic approach or species-specific differences [24,37].

Table 2 summarizes proteins identified in oEVs in mouse and human and the 25 most abundant in bovine. As Table 2 shows, only two proteins overlap among species, heat shock protein family A (Hsp70) member 8 (HSPA8) and actin beta (ACTB). This could be mainly due to the different approaches and methodologies used in bovine, murine and human studies (focusing on specific proteins by Western Blotting versus extensive analysis of oEVs proteins by mass spectrometry). It is worth mentioning that HSPA8, is a protein commonly used as exosomal marker but also a protein with a key role in the oviduct. It has been identified in a subset of 70 kDa oviductal surface proteins that bound to spermatozoa. Different roles have been attributed to this protein: mediating oviduct-gamete interactions, enhancing survival of spermatozoa, and improving sperm fertilizing ability [73,74].

### 3.2. Oviductal EVs and mRNA

Our recent study analysing the oEVs mRNA content across the estrous cycle identified a high number of transcripts corresponding to 13,197 genes [37]. However, most of the transcripts occurred at low a copy numbers and only the top 500 most abundant transcripts showed a frequency of more than 25 transcripts per million. We set this arbitrary threshold to point out oEVs transcripts that may exert a functional effect on spermatozoa and embryos, since we hypothesize that mRNA content at low concentration is less likely to induce an effect and to distinguish from possible contaminations on EVs pellet as a result of the purification method. Among the identified mRNAs, mRNAs encoding ribosomal proteins and translation elongation factors were the most abundant protein-coding RNAs in oEVs. Ribosomal proteins play a crucial role in protein translation, immune signalling and development [75]. It is possible that mRNAs encoding ribosomal proteins, translation factors as well as tRNAs and rRNAs, contained in oEVs, may participate in protein translation upon arrival to embryos.

Functional analysis of oEVs content showed that oEVs were enriched in mRNA associated to embryo development, cell proliferation and epigenetic regulation. We identified an important number of genes involved in chromatin modification including histone methyltransferases (*EHMT1, EHMT2, EZH1, KMT2A, KMT2B, KMT2C, PHF1, PHF2, PRMT5, SETD1A, SETD2*) and histone demethylases (*ARID5B, JMJD1C, KDM2A, KDM3B, KDM5B, KDM5C, KDM6B*), but also one DNA methyltransferase gene (*DNMT1*). This finding suggests that chromatin modification in the early embryo could be in part under maternal control via oEVs transcripts. Moreover, considering that altered DNA methylation patterns are induced in the embryo by suboptimal culture conditions [76], we hypothesize that oEVs supplementation during in vitro culture could revert in parts those epigenetic modifications in the pre-implantation embryo.

Pointing out at specific mRNA components of oEVs with potential roles on gametes/ embryos, tumour protein translationally-controlled 1 (*TPT1*) was the most frequent mRNA identified in oEVs, which has been associated with the activation of pluripotency genes such as *NANOG* and *OCT4* in oocytes [77]. The protein encoded by another mRNA highly abundant in oEVs, receptor of activated protein kinase C 1 (RACK1) has been found in higher abundance in bovine blastocysts compared to morula [78]. We also identified in oEVs genes involved in transcription and translation (*CNOT1, EEF1G, PABPN1, FOXO3A, DOT1L*) and in metabolism (*GALE, HEBP1*) which has been found suppressed in in vitro embryos compared to in vivo ones [79]. Different mRNAs for CATSPER units, which are involved in sperm Ca^2+^ channels and associated to sperm fertility were also identified in oEVs.

Nakano et al., [60], showed that vimentin, *GAPDH* and *ACTB* mRNAs are also components of murine oEVs isolated from oviductal mesenchymal cell line (S1), which were also identified in bovine oEVs in our study. Moreover, they demonstrated that these specific mRNAs from an oviductal mesenchymal cell line were able to be transported into cells of Müllerian epithelial cell line (E1) via oEVs and exert a functional effect by increasing the number of ciliated cells. On the other hand, Lopera-Vásquez et al. [23] showed an upregulated expression of connexin 43 (*GJA1*) and also *GAPDH* in in vitro produced embryos after oEVs supplementation during culture. Although these authors did not analyze the EVs content, our study [37] identified *GJA1* and *GAPDH* mRNAs in oEVs, which are associated with better quality embryos and cryotolerance [80].

### 3.3. Oviductal EVs and miRNA

So far, only two publications have performed an extensive analysis of miRNA in oEVs across the estrous cycle, identifying 272 miRNAs and 184 miRNAs in murine and bovine oEVs, respectively [31,37]. Both studies showed a hormonal effect of the estrous cycle; however, the number of differentially miRNAs among stages was not very high in either study. Fereshteh et al. [31] suggested that miRNAs expression levels did not appear to vary much throughout the estrous cycle, which may be due to the mixture of oEVs from stage 1–2 and stage 3–4 in their study. However, Almiñana et al. [37] analysed the four stages separately and did not find a high number of differential miRNAs among stages (13 out of 184 identified). Nevertheless, great differences where observed at mRNA, proteins and other non-coding RNA such as small nuclear RNAs in the same samples of oEVs [37].

Comparing miRNAs identified in oEVs from murine (272) [31] and bovine (184) [37] studies, we found that 91 miRNAs were in common, while 93 were unique to bovine and 183 to murine oEVs. These results may point out common and species-specific molecular components of oEVs, which may participate in the gamete/embryo-oviduct cross-talk in a species-specific manner. Since monotocous and polytocous species have shown differences in gamete/embryo maternal interactions [81]. However, it is worth mentioning that some differences in miRNAs content could also be due to the use of different oEVs isolation protocols and differences in RNA-sequencing analysis (mainly small RNA library preparation [82]). Moreover, it is important to consider annotation issues, since more miRNAs are known for mice than for cattle. Additionally, differences in miRNAs annotation according to the species make it difficult to perform direct comparisons among species and can mislead the numbers of common or specific miRNAs in each species.

In canine oEVs, three miRNAs have been identified by Consiglio et al. [34] (miR-30b, miR-375 and miR-503), particularly in MVs from canine oviductal fluid with key roles in follicular growth and oocyte maturation. Two of them (miR-30b and miR-375) were also present in bovine and murine oEVs miRNA [31,37]. Interestingly, miR-375 associated to amino sugar and nucleotide sugar metabolism [37] and involved in cell fate determination in the early embryo [83], and it was significantly different among stages of the estrous cycle (stage 3 compared to stage 1) in the study of Almiñana et al. [37].

**Figure 1:**
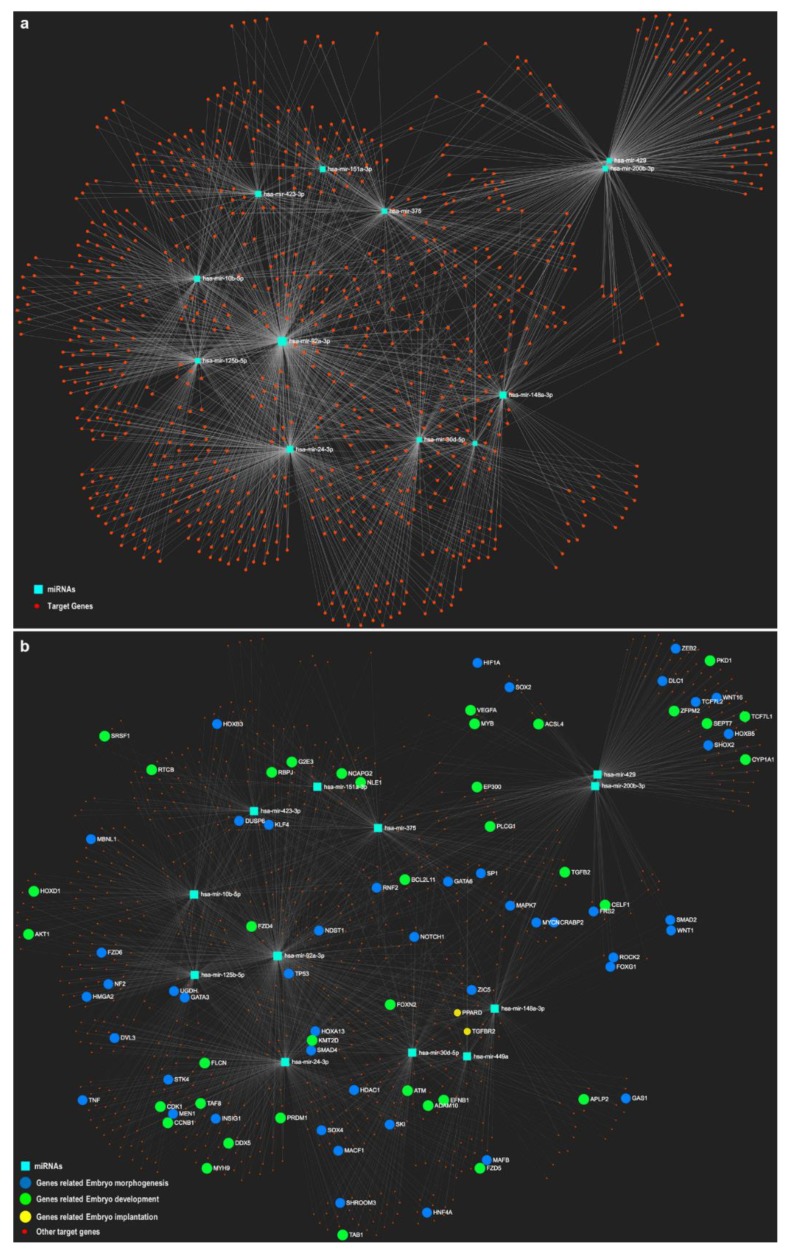
Networks of selected miRNAs identified in oviductal extracellular vesicles (oEVs) and potential target genes involved in embryo development. The miRNet online tool was used to represent selected miRNAs (13) (turquoise-blue nodes) based on differential expression and high abundance in oEVs at stage 1 of the bovine estrous cycle (recently ovulated follicle; days 1–4 post-ovulation) and potential target genes (red nodes) enriched in functional categories related to embryo development (green), embryo morphology (blue) and implantation (yellow) in a network. Since the miRNet online tool allows to choose the source/tissue for the list of miRNAs, network (**a**) represents 11 miRNAs contained in exosomes in the online tool database, with all target genes involved in embryo development. Network (**b**) represents 3 miRNAs contained in embryos in the online tool database, with all target genes involved in embryo development functions.

**Figure 2:**
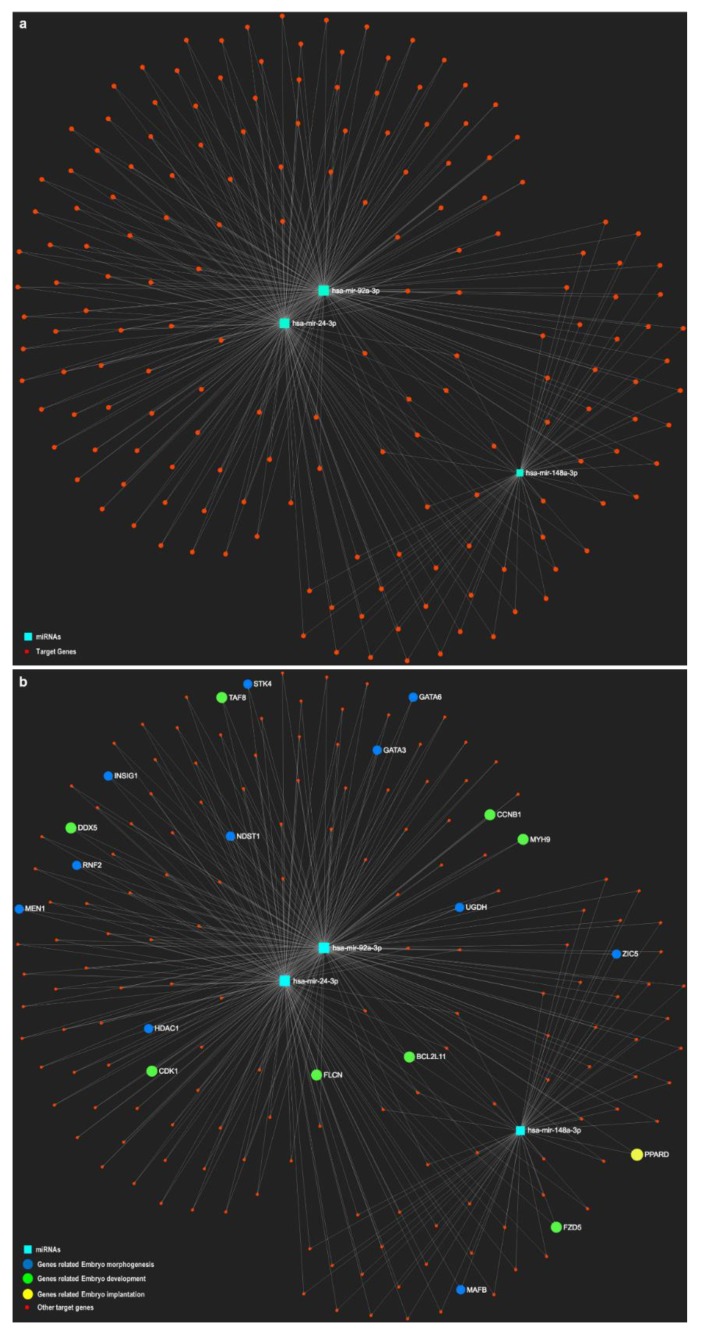
Networks of selected miRNAs identified in oviductal extracellular vesicles (oEVs) and contained in the embryo according to miRNet online tool with potential target genes involved in embryo development. The miRNet online tool was used to represent selected miRNAs (13) (turquoise-blue nodes) based on differential expression and high abundance in oEVs at stage 1 of the bovine estrous cycle (recently ovulated follicle; days 1-4 post-ovulation) and potential target genes (red nodes) enriched in functional categories related to embryo development (green), embryo morphology (blue) and implantation (yellow) in a network. Since the miRNet online tool allows to choose the source/tissue for the list of miRNAs, the network (**a**) represents three miRNAs contained in embryos according to the online tool database, with all target genes involved in embryo development functions (**b**).

In an attempt to provide a comprehensive analysis of oEVs-derived miRNAs and their potential target genes in embryos, we used the miRNet online tool, an integrated platform linking miRNA targets and functions (https://www.mirnet.ca/) [84], with lists of miRNAs differential and highly abundant in bovine oEVs [37]. This analysis revealed that a considerable number of miRNAs contained in oEVs target genes involved in embryo development, embryo morphology or implantation as represented in the networks of Figure 1 and Figure 2. Since the miRNet online tool allows to choose the source/tissue for the list of miRNAs, we selected miRNAs contained in exosomes for the network in Figure 1 and in embryos for Figure 2. Figure 1 shows that 11 out of 13 miRNAs were present in EVs in the online tool database (Figure 1a), with all of them targeting genes involved in embryo development (Figure 1b). Figure 2 shows that only 3 miRNAs found in oEVs were also found in embryos according to the miRNet database (Figure 2a) and target also genes involved in embryo development (Figure 2a). In a similar way, Fereshteh et al. [31] showed that oEVs miRNA target genes involved in embryo development including *BCL2*, *CDK6* and c*-MYC* [85,86]. 

Regarding functional studies of oEVs-derived miRNAs with gametes, Fereshteh et al. [31] showed recently that murine oEVs can deliver miRNAs to sperm, particularly: miR-143-3p, miR-22-3p, and miR-34c-5p, which are associated to reproductive processes such as embryo implantation, spermatogenesis and the early zygote [85,87]. Individual miRNAs were predominantly localized in specific head compartments, with miR-34c-5p being highly concentrated at the centrosome. Interestingly, miR-34c-5p, is essential for the first cleavage and is solely sperm-derived in the zygote. MiR-34c-5p was also identified in bovine oEVs [37] and could have been taken up by embryos after oEVs incubation during IVC [24], but so far this has not been shown. For miR-30d contained in oEVs and also in uterine EVs, there is evidence for uptake by mouse embryos [88]. The results of Fereshteh et al. [31] show that oEVs are capable of contributing to the sperm’s miRNA repertoire and exert an effect with physiological relevance to the sperm.

### 3.4. Oviductal EVs and other Molecular Cargo

Lipids, other small molecules and metabolites are also essential components of EVs [89,90]. To the best of our knowledge, no lipidomic or metabolomic analyses of oEVs have been performed so far. Having a look into the literature, it is not surprising since not many metabolomics studies have been published in the field of female reproduction. The metabolomic profiling of the bovine oviductal fluid across the estrous cycle has been performed recently [91]. While lipidomic studies on the oviduct have focused only on analysing specific lipids in a short time window after ovulation (day 4) [92]. Furthermore, metabolomic investigations were focused on the embryo culture medium with the idea to find biomarkers for embryo selection or on the follicular fluid to predict oocyte quality [93,94]. 

Although, small molecules of oEVs have not been studied yet, results of exosome from other sources suggest potential functions of these molecules also in the maternal-gamete/embryo communication. For example, recent studies have shown that exosomes derived from immune cells and other cells contain bioactive lipids, such as prostaglandins, leukotrienes, and lysophospholipids and may transfer them to specific target cells (reviewed in [95]). Given the importance of prostaglandins in reproductive processes [96], targeted transport of prostaglandins to the early embryo or from the embryo to oviduct epithelial cells could be possible.

Overall, the characterization of the oEVs metabolomic and lipidomic profile could allow the identification and quantitation of an extensive variety of molecules indicative of metabolic, nutritional as well as the physiological and pathological status of the cells of origin, animal or patient. These valuable data will complement our current knowledge about the oEVs at transcriptomic and proteomic level, increasing our understanding of the potential role of oEVs during the early reproductive events. Moreover, the exosomal lipid composition is essential for the formation and release of exosomes [97], which is an important point to consider when we aim to use EVs/exosomes as drug delivery vehicles. 

### 3.5. Regulation of the Dynamic and Complex Oviductal EVs Molecular Cargo

Aside from the extensive identification of oEVs molecular components, our laboratory provided with clear evidence that the oEVs content is under hormonal regulation with marked differences between the pre-ovulatory stage and post-ovulatory stages of the estrous cycle at mRNA, protein and small noncoding RNA level [37]. This agrees with the results of transcriptome and proteome studies of the oviduct during the estrous cycle [16,98,99,100,101]. Furthermore, Greening and colleagues [102] showed that the EVs protein cargo derived from a human endometrial cell line was also under hormonal regulation. Furthermore, Burns et al. [103] recently showed that exogenous progesterone treatment (P4) regulates uterine EVs production and their mRNA and miRNA cargo in the uterine lumen of sheep. Additionally, these authors demonstrated that the molecular content varies between pregnant and cyclic animals by showing differential protein abundance in uterine EVs collected from day 14 pregnant sheep versus cyclic controls [104]. These findings suggest differential loading of EVs components derived from the endometrial epithelia or from the conceptus. Moreover, these results support the idea that uterine EVs play a key role in conceptus-endometrial interactions, crucial for the establishment and maintenance of pregnancy.

**Figure 3:**
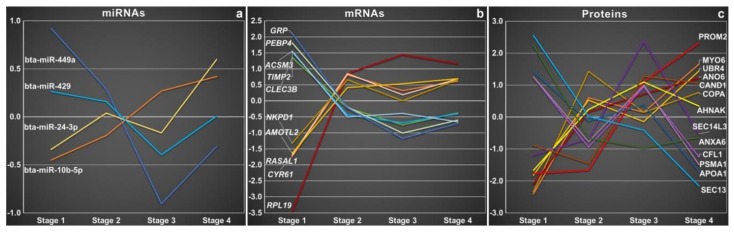
Profiles of miRNAs, mRNAs, and proteins during the estrous cycle in bovine oviductal extracellular vesicles (oEVs). MicroRNAs (**a**), mRNAs (**b**), and proteins (**c**) showing the most pronounced and representative changes with respect to typical expression profiles during the estrous cycle in oEVs were selected. Similar profiles are shown in related colors. Relative expression is shown as mean-centered expression values (log2-transformed expression value of the stage minus mean of all 4 stages). Stage 1: recently ovulated follicle (days 1–4 post-ovulation (po)); stage 2: early luteal development with medium or large follicles or both present (days 5–11 po); stage 3: fully functional corpus luteum (CL) yellow or orange in color (days 11–17 po); stage 4: regressing CL with little vasculature and a large preovulatory follicle present (days 18–20 po).

A more detailed analysis of the hormonal influence of the estrous cycle on selected components of oEVs cargo, provided a very dynamic and complex profile of proteins, mRNAs and miRNAs (Figure 3). The miRNAs, mRNAs and proteins represented in Figure 3 showed the most pronounced and representative changes with respect to typical expression profiles during the estrous cycle in oEVs in our study, mainly with marked differences between post- and pre-ovulatory stage (stage 1 and stage 4). At the miRNA level, two different abundance patterns for 4 selected miRNAs were found (Figure 3a). While bta-miR-24-3p and bta-miR-10b-5p were downregulated in stage 1 and upregulated in stage 4, bta-miR-429 and bta-miR-449a concentration was highest in stage 1 and lowest for stage 3. At mRNA level, biggest differences in mRNA abundance were found in stage 1 compared to the other stages, being some mRNAs upregulated while others downregulated in stage 1 (Figure 3b). In contrast, the mRNA abundance was more or less stable across the rest of the stages analysed (stage 2–4). Some proteins showed a biphasic pattern of abundance with highest concentrations in stages 1 and 3 (see Figure 3c). These findings show a differential loading of oEVs content in the oviduct across the estrous cycle and highlight the biggest differences at the postovulatory stage (stage 1, 0–4 days after ovulation), the time for arrival of gametes, fertilization and early embryo development.

Having these results in mind, and with the idea of performing further studies on the oEVs cargo under different reproductive scenarios, we would like to mention some of our concerns that may be also applied to other reproductive studies: (1) Samples from our study were collected from abattoir animals and classified according to the ovary status [37], a more accurate classification using synchronized animals and collecting samples for a specific day of the cycle could reveal slightly different results; (2) The results for one specific stage are the mean of abundance of all samples collected between different days (for example for stage 1, from day 1-4 post-ovulation), it is possible that there are also differences among days in this short window of time; (3) Samples from animals under different nutritional, housing (e.g., temperature, stress), animal management conditions or other factors such as animal age or breed can also affect the EVs population and cargo; (4) To obtain a considerable amount of EVs for omics studies, oEVs from our study represent a mixture of the two oviducts of the same animal. It is possible that there are differences in the oEVs population and content between ipsi-and contralateral oviducts and also between regions of the oviduct ampulla and isthmus, as Lopera-Vásquez results suggested [59]. These authors pointed out that the isthmus had a higher concentration of smaller vesicles compared to the ampulla.

Considering the great hormonal effect on the oEVs cargo observed in our study, we raise the question whether the arrival of spermatozoa and/or embryos into the oviduct could also modulate the oEVs molecular components. Previous studies have shown that the OEC and oviductal fluid alter their composition in response to sperm and/or embryos [4,6,7]. As oEVs are major components of OF, we hypothesize that the oEVs may follow a similar pattern as a part of the oviductal response to gametes/embryos to support the success of the early reproductive events. Furthermore, it raises the question if oEVs may also differ under conditions of infertility or repeated pregnancy failure, in the present of unhealthy gametes/embryos. Further investigations determining the secretion/release of oEVs under different reproductive scenarios or conditions will bring some light to this important matter.

## 4. Oviductal EVs and Their Potential as Therapeutic Vectors

The increasing number of reviews published in the last five years on the role of EVs in reproduction, underlines their importance in the early reproductive events, and also highlights their potential as therapeutic vectors and diagnostic tools in ARTs [26,28,29,30,105,106,107,108,109]. However, only a very few studies have provided molecular proofs of the role of EVs in the oviduct that promote their use to improve ARTs outcomes. In this review, we drew together findings from oEVs studies focusing on the oEVs components at protein, mRNA or miRNA level that may modulate the dialogue between the gametes/embryo and the oviductal environment. The transfer of proteins from the oviduct to sperm/embryos via oEVs may help sperm, in the decapacitation/capacitation events and promoting fertilizing ability [22,32], while for embryos, oEVs may support the early embryo development processes [24]. The transfer of mRNAs could lead to the generation of functional proteins by the embryo while transferring genetic information. The transfer of miRNAs could have a remarkable impact on the regulation of gene expression. Altogether, these findings suggest oEVs represent ideal natural nanoshuttles for carrying a “cocktail” of proteins, transcripts and miRNAs that are not present in the in vitro culture of gametes and embryos, or are altered after gametes/embryos are subjected to sperm handling and ARTs procedures.

In this regard, on the sperm side, studies have shown differences in transcript abundance in sperm after cryopreservation [110], and particularly, differences in miRNAs in fertile versus subfertile [111] and low versus high motile, cryopreserved sperm in bulls [112]. The transfer of specific transcripts into sperm have important implications in fertility, since some sperm RNAs are delivered into the zygotes and regulate cell signaling processes or regulate epigenetic events during early embryo development [110]. On the embryo side, comparative studies regarding the quality of embryos derived from ART versus in vivo embryos have shown important differences in the embryonic transcriptome and proteome profiles. For example, the suppressed expression of genes involved in transcription and translation (*CNOT1*, *EEF1G*, *PABPN1*, *FOXO3A*, *DOT1L*) and metabolism (*GALE*, *HEBP1*) [79] and reduced expression of annexins 1 and 2 proteins in IVP embryos, with important functions in the maintenance of placentation [113]. Considering these findings, the use of oEVs seems a good strategy to overcome in vitro culture deficiencies and promote successful pregnancy when ARTs are applied. 

For the practical application of oEVs, different sources of oviductal secretions can be considered as a starting material, for example: in vivo oviducts, when it is possible, or in vitro conditioned media obtained from primary culture of OEC or immortalized OEC cell lines. Caution should be taken when in vitro material is used, since differences in protein composition have been shown between in vivo and in vitro oEVs [37]. Being aware of these differences, oEVs could be loaded by co-incubation or electroporation with specific components (proteins, miRNA) that are not present in in vitro, or with components that could exert a specific effect in gametes/and embryos.

Then, oEVs could be applied to: (a) sperm: to bring decapacitation factors during sperm-pre-incubation or washing steps in ARTs procedures or after cryopreservation, since it is known that sperm handling procedures and storage induce sperm surface changes and damage with important consequences for sperm survival and fertilization competence [114]; (b) oocytes: to improve in vitro maturation rates, as shown by Lange-Consiglio et al. [34] or to improve blastocyst yield, since the intrinsic quality of the oocyte is the main factor affecting blastocyst yield [115]; (c) embryos, during IVC to improve embryo development and quality, as recent studies have shown [23,24,36], since the post-fertilization culture conditions constitute the main factor affecting the quality of the blastocyst [116]; and also by supplementing ET media, to improve pregnancy and birth rates [36].

## 5. Challenges and Future Directions for oEVs Research

Research in the field of reproductive EVs has increased exponentially in the last decade. More recent investigations are focusing on the role of oEVs in the early reproductive events. These studies clearly show that oEVs play a crucial role in sperm capacitation and fertilizing ability, oocyte maturation, fertilization and early embryo development, which are the first crucial events for pregnancy success. It furthermore calls for a better understanding of the molecular mechanisms mediating the gamete/embryo-oviductal cross talk, before oEVs research can be translated to therapeutic approaches to improve the outcomes of natural pregnancy and increase pregnancy rates in ARTs in livestock and humans.

As discussed throughout this review, future efforts should be directed to: (1) standardization of isolation and purification methods for EVs that can be adapted for oEVs, of which the availability and quantity is very limited compared to other reproductive EVs; (2) well-defined markers for the characterization of heterogeneous EVs subpopulations in the oviduct (exosomes and MVs); which will allow comparisons among studies; (3) deep analyses of oEVs molecular cargo under physiological and pathological conditions using advanced proteomic and sequencing technologies, which is crucial to identify the targets in the potential recipient cells (gametes/embryo); (4) this should come along with functional studies, which will help to understand the molecular signalling networks triggered by oEVs in gametes/and embryos; (5) studies on specific mechanism of oEVs uptake by sperm, oocyte and embryo (recognition of oEVs and entering into the cell); (6) studies on cargo delivery, i.e., what is released into the cell and what is the destiny of the delivered molecules; and (7) finally, but no less important, defining the extent of the oEVs effects in the reproductive events, from gametes and embryos to the delivery of healthy descendants and their impact on adulthood. 

Considering the work ahead, the therapeutic application of oEVs outlined above seems far away. However, recent guidelines published by ISEV about developing best practice models for EVs therapeutic use and their application in clinical trials [117,118], together with the number of early-phase clinical trials with EVs that have been already undertaken (reviewed by [119]), shows that progress is being made towards promoting safe approaches for clinical therapeutic applications of EVs [120]. Future research facing current challenges in reproductive EVs and particularly in oEVs, will ensure advances in the potential application of oEVs to maximize pregnancy rates in natural and in in vitro fertilization treatments.

## 6. Concluding Remarks

In this review, we attempted to bring together current knowledge of EVs in the oviductal environment, from isolation, characterization, description of the EVs molecular content and associated functional aspects in different species. The potential role of oEVs as modulators of gamete/embryo-oviduct interactions and their implications in the success of the early reproductive events has been addressed. Our review points out current challenges in oEVs research, emphasizing the need for further descriptive and functional research on oEVs under different physiological and pathological conditions. This will allow to set directions for the potential application of oEVs as therapeutic vectors to improve the diagnosis and treatment of pregnancy disorders, infertility problems and increase the success of ARTs.

## Figures and Tables

**Table 1:** Summary of published studies related to oviductal extracellular vesicles (oEVs).

Topic	Species	Year	Findings	Analyzed oEVS Content	Characterization Method	Exosomal Molecular Markers	Citation
**Analysis of oEVs molecular content**	Bovine	2017	Identification of proteins from bovine oEVs cargo from in vivo and in vitro origin. Oviductal EVs carry proteins associated to sperm-binding, fertilization and embryo development.	Proteins	TEM and WB	HSP70 (WB)	Almiñana et al., 2017 Reproduction [24]
	Bovine	2018	Identification of mRNAs, small ncRNAs and proteins from bovine oEVs cargo across the estrous cycle. Biological relevance of oEVs content on sperm/embryo and implications for the reproductive success.	proteins, mRNA and small ncRNA	TEM and WB	HSP70 and ANXA1 (WB)	Almiñana et al., 2018 BMC Genomics [37]
	Murine	2018	Identification of miRNAs from murine EVs cargo across the estrous cycle. Biological relevance of miRNA derived from murine oEVs on sperm and embryo.	miRNAs	TEM and WB	CD9 (WB)	Fereshteh et al., 2018 Scientific Reports [31]
**oEVs and their functional effects on gametes (sperm and oocyte)**	Murine	2013	Identification of EVs in the murine oviduct for first time and characterization. Expression of Plasma Membrane Ca^2+^ ATPase 4a (PMCA4a) in the Murine female tract during estrous cycle and in oEVs: role in hyperactivated motility and fertility. Transport of PMCA4a from oviduct to sperm: PMCA4a uptake by sperm via oEVs.	protein PMCA4a	TEM and WB	HSC70 and CD9 (WB); CD9 (TEM)	Al-Dossary et al., 2013 PLOS One [22]
	Murine	2015	Oviductosome-Sperm Membrane Interaction in Cargo Delivery: Detection of Fusion and Underlying Molecular Players Using Three-Dimensional Super-Resolution Structured Illumination Microscopy (SR-SIM).	No	TEM and WB	PMCA4 (TEM) CD9 (WB)	Al-Dossary et al., 2015 J Biol Chem [58]
	Avian	2017	Identification of oEVs from culture medium of Utero-vaginal junction and vagina cells from hens: Changes in localization and density of CD63-positive exosome-like substances in the hen oviduct. Effect of oEVS on avian sperm viability and motility.	No	WB	CD63 (WB)	Huang et al., 2017 Theriogenology [33]
	Canine	2017	Identification of oviductal MVs from canine oviductal epithelial cells culture and characterization. Oviductal MVs improve in vitro maturation of canine oocytes. Oviductal MVs contain miRNAs (miR-30b, miR-375 and miR-503) with roles in follicular growth and oocyte maturation.	hsa-miR-30b, has-miR-375, cfa-miR-503	NTA	_	Lange-Consiglio et al., 2017 Reproduction [34]
	Turtle	2017	Identification of oEVs from turtle oviducts and characterization. Oviductal EVs contact with cilia and with the sperm membrane give this turtle a unique secretory morphology.	No	TEM and immunostaining	CD63 (Immunostaining)	Waqas et al., 2017 J. Exp. Zool. [35]
	Murine	2018	Evidence of delivery of miRNA contained in oEVs to sperm. Localization of miiR-34c-5p at the centrosome in the sperm after uptake.	miRNAs	TEM and WB	CD9 (TEM) CD9 (WB)	Fereshteh et al., 2018 Sci Rep [31]
	Human/Murine	2018	Identification of EVs in the woman fallopian tubes for first time and characterization. Oviductal EVs their fertility-modulating proteins are conserved in humans. PMCA1 and PMCA4 in oEVS are enzymatically active and their activity increased in sperm after oEVs interaction.	proteins (PMCA1-4)	TEM anand WB	PMCA4 (TEM) HSC70 (WB)	Bathala et al., 2018 Mol Hum Reprod [32]
**oEVs and their functional effects on the embryo(s)**	Bovine	2016	Oviductal EVs from in vitro origin (BOEC) improve embryo quality (number/cells and gene expression) and cryosurvival.	No	TEM, NTA and FC, WB	CD9 and CD63 (FC) CD9, ERM and TSG101 (WB)	Lopera-Vásquez et al., 2016 PLOS One [23]
	Bovine	2017	Embryos take oEVs up during in vitro culture. Oviductal EVs improve embryo development and embryo quality.	proteins	TEM and WB	HSP70 (WB)	Almiñana et al., 2017 Reproduction [24]
	Bovine	2017	Identification and characterization oEVS from different regions of oviduct (ampulla and isthmus): differences in size and concentration of oEVS. Oviductal EVs from ampulla and isthmus have different effect on in vitro embryo development: oEVs- isthmus derived induce a positive effect on embryo quality.	No	TEM, NTA and WB	CD9, ERM and TSG101 (WB)	Lopera-Vásquez et al., 2017 Reproduction [59]
	Murine	2019	Differences in oEVs from murine donor and recipient oviductal fluid: donor higher concentrations and more protein than recipients. Oviductal EVs from donors improve the efficiency of embryo transfer: improve birth rate via resisting apoptosis and promoting differentiation.	No	TEM; NTA; WB; BCA	CD9 and HSP70 (WB)	Qu et al., 2019 Reproduction, Fertility and Development [36]
**Other oEVs functions**	Murine	2017	Role of oEVs in the interaction between epithelial and mesenchymal cells: oEVs derived from mesenchymal cells modulate the oviductal ciliogenesis.	mRNA (beta-actin, GAPDH and Vimentin)	TEM and WB	CD9 and CD81(WB) C81 (TEM)	Nakano et al., 2017 Biochem & Biophys Res Com [60]

TEM: transmission electron microscopy; NTA: nanoparticle tracking analysis; WB: Western blotting; FC: flow cytometry; BOEC: bovine oviductal epithelial cells; MVs: microvesicles; BCA: protein assay by bicinchoninic acid. All studies used ultracentrifugation as method to isolate oEVs, except for oEVs from turtle (Waqas et al., 2017), in which oEVs were not purified and were identified by immunohistochemistry and TEM.

**Table 2:** Proteins identified in oviductal extracellular vesicles (oEVs) in different species.

Bovine	Murine		Human		
Protein Description	Symbol	Protein Description	Symbol	Protein Description	Symbol
oviductal glycoprotein 1	OVGP1	Plasma membrane calcium-transporting ATPase 4	PMCA4	Plasma membrane calcium-transporting ATPase 4	PMCA4
annexin A1	ANXA1	Plasma membrane calcium-transporting ATPase 1	PMCA1	Plasma membrane calcium-transporting ATPase 1	PMCA1
tubulin, beta 2B class IIb	TUBB2B	Endothelial nitric oxide synthase	eNOS (NOS3)	Endothelial nitric oxide synthase	eNOS (NOS3)
annexin A2	ANXA2	neuronal nitric oxide synthase	nNOS (NOS1)	neuronal nitric oxide synthase	nNOS (NOS1)
annexin A4	ANXA4	calcium/calmodulin-dependent serine kinase	CASK	calcium/calmodulin-dependent serine kinase	CASK
heat shock protein family A (Hsp70) member 8	HSPA8	heat shock protein family A (Hsp70) member 8	HSPA8 (HSC70)	heat shock protein family A (Hsp70) member 8	HSPA8 (HSC70)
actin beta	ACTB	actin beta	ACTB	actin beta	ACTB
CD109 molecule	CD109				
tubulin, alpha 3e	TUBA3E				
annexin A5	ANXA5				
heat shock 70kDa protein 1A	HSPA1A				
heat shock protein 90 alpha family class A member 1	HSP90AA1				
5’-nucleotidase ecto	NT5E				
annexin A8-like 1	ANXA8L1				
ezrin	EZR				
clathrin heavy chain	CLTC				
glyceraldehyde-3-phosphate dehydrogenase	GAPDH				
stomatin	STOM				
mesothelin	MSLN				
major vault protein	MVP				
annexin A11	ANXA11				
ectonucleotide pyrophosphatase/phosphodiesterase 3	ENPP3				
heat shock protein family B (small) member 1	HSPB1				
clusterin	CLU				
RAB5C, member RAS oncogene family	RAB5C				

Bovine: Only the 25 most abundant proteins are shown in the table. A complete list of the bovine oEVS proteins identified by Mass Spectrometry so far can be found in Almiñana et al., 2018. Murine: Murine oEVs proteins have been identified by Western blot immunolabeling (source: Bathala et al., 2018). Human: Human oEVs proteins have been identified by Western blot or immunolabeling (source: Bathala et al., 2018).
